# Laparoscopic parenchyma-sparing liver resection for large (≥ 50 mm) colorectal metastases

**DOI:** 10.1007/s00464-022-09493-3

**Published:** 2022-08-03

**Authors:** Davit L. Aghayan, Gabriella d’Albenzio, Åsmund A. Fretland, Egidijus Pelanis, Bård I. Røsok, Sheraz Yaqub, Rafael Palomar, Bjørn Edwin

**Affiliations:** 1 The Intervention Centre, Oslo University Hospital - Rikshospitalet, 0027 Oslo, Norway; 2 Department of Surgery N1, Yerevan State Medical University After M. Heratsi, Yerevan, Armenia; 3 Department of Informatics, University of Oslo, Oslo, Norway; 4 Institute of Clinical Medicine, Medical Faculty, University of Oslo, Oslo, Norway; 5 Department of HPB Surgery, Oslo University Hospital - Rikshospitalet, Oslo, Norway; 6 Department of Computer Science, Norwegian University of Science and Technology, Gjøvik, Norway

**Keywords:** Laparoscopy, Liver resection, Large tumors, Parenchyma-sparing

## Abstract

**Background:**

Traditionally, patients with large liver tumors (≥ 50 mm) have been considered for anatomic major hepatectomy. Laparoscopic resection of large liver lesions is technically challenging and often performed by surgeons with extensive experience. The current study aimed to evaluate the surgical and oncologic safety of laparoscopic parenchyma-sparing liver resection in patients with large colorectal metastases.

**Methods:**

Patients who primarily underwent laparoscopic parenchyma-sparing liver resection (less than 3 consecutive liver segments) for colorectal liver metastases between 1999 and 2019 at Oslo University Hospital were analyzed. In some recent cases, a computer-assisted surgical planning system was used to better visualize and understand the patients’ liver anatomy, as well as a tool to further improve the resection strategy. The surgical and oncologic outcomes of patients with large (≥ 50 mm) and small (< 50 mm) tumors were compared. Multivariable Cox-regression analysis was performed to identify risk factors for survival.

**Results:**

In total 587 patients met the inclusion criteria (large tumor group, *n* = 59; and small tumor group, *n* = 528). Median tumor size was 60 mm (range, 50–110) in the large tumor group and 21 mm (3–48) in the small tumor group (*p* < 0.001). Patient age and CEA level were higher in the large tumor group (8.4 μg/L vs. 4.6 μg/L, *p* < 0.001). Operation time and conversion rate were similar, while median blood loss was higher in the large tumor group (500 ml vs. 200 ml, *p* < 0.001). Patients in the large tumor group had shorter 5 year overall survival (34% vs 49%, *p* = 0.027). However, in the multivariable Cox-regression analysis tumor size did not impact survival, unlike parameters such as age, ASA score, CEA level, extrahepatic disease at liver surgery, and positive lymph nodes in the primary tumor.

**Conclusion:**

Laparoscopic parenchyma-sparing resections for large colorectal liver metastases provide satisfactory short and long-term outcomes.

**Graphical abstract:**

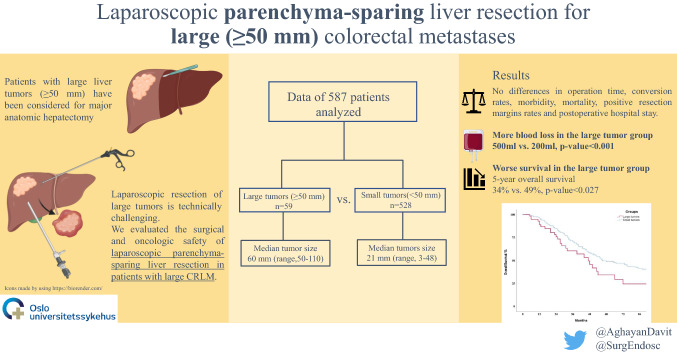

Minimally invasive procedures have revolutionized the surgical practice in many surgical sub-specialties as well as in hepatobiliary surgery. Laparoscopic liver surgery has shown its numerous advantages over conventional open surgery and has been established as a first-line surgical approach in specialized centers, despite its relatively slow implementation [1–4].

Over the last two decades, the evidence level of laparoscopic liver surgery has increased significantly, from small case series of selected patients to large multi-center series and randomized control trials [5–7]. This minimally invasive liver surgery has been well reported for benign and malignant liver tumors, including primary and secondary liver malignancies [8–11]. In 2017 the European consensus guidelines meeting for laparoscopic liver surgery held in Southampton, United Kingdom, it was advocated that the laparoscopic approach should be considered standard practice for lesions in the left lateral and the anterior segments, while technically challenging resections, such as repeated resections or 2-staged hepatectomies, resections for large lesions, and lesions close to the liver hilum were considered possible by surgeons with extensive experience in laparoscopic liver surgery[12]. Earlier, in the first international consensus meeting (the Louisville Statement, 2008), it was stated that the patients with solitary lesions, 50 mm or less, located in the antero-lateral segments are acceptable indications for laparoscopic liver resection [13].

In our center, the main indication for laparoscopic liver resections is colorectal liver metastases (CRLM), where the parenchyma-sparing strategy is the method of choice [1, 14, 15]. However, the laparoscopic parenchyma-sparing approach to resect large lesions is challenging, and careful pre-operative surgical planning is essential for evaluating the chosen resection strategy. In this context, the use of computer-assisted resection planning systems can provide surgeons with an accurate characterization of the resection in terms of trajectory, safety margins, and resection volumetry [16]. To the best of our knowledge, most of the studies on laparoscopic parenchyma-sparing liver resections (LPSLR) reported the results of single small metastases. Earlier, we reported our experience in LPSLR for patients with multiple CRLM and metastases located in the postero-superior liver segments [17, 18]. The current analysis aimed to evaluate the surgical and oncologic outcomes after LPSLR in patients with large (≥ 50 mm) CRLM.

## Methods

### Study design and definitions

The study was conducted at Oslo University Hospital, a tertiary referral center for hepato-pancreato-biliary surgery for South-Eastern Norway Health Authority, serving about three million population. Patients who primarily underwent laparoscopic parenchyma-sparing (defined as a resection of less than three consecutive liver segments) liver resection for colorectal liver metastases between 1999 and 2019 at Oslo University Hospital were identified from the prospectively registered database and included in this study. Patients that had previously undergone liver resection were excluded. The surgical and oncologic outcomes of patients with large (≥ 50 mm) and small (< 50 mm) tumors were retrospectively analyzed and compared. The Institutional Review Board approved the study and due to the retrospective nature of the study written consents from the patients was not required.

**Fig. 1:**
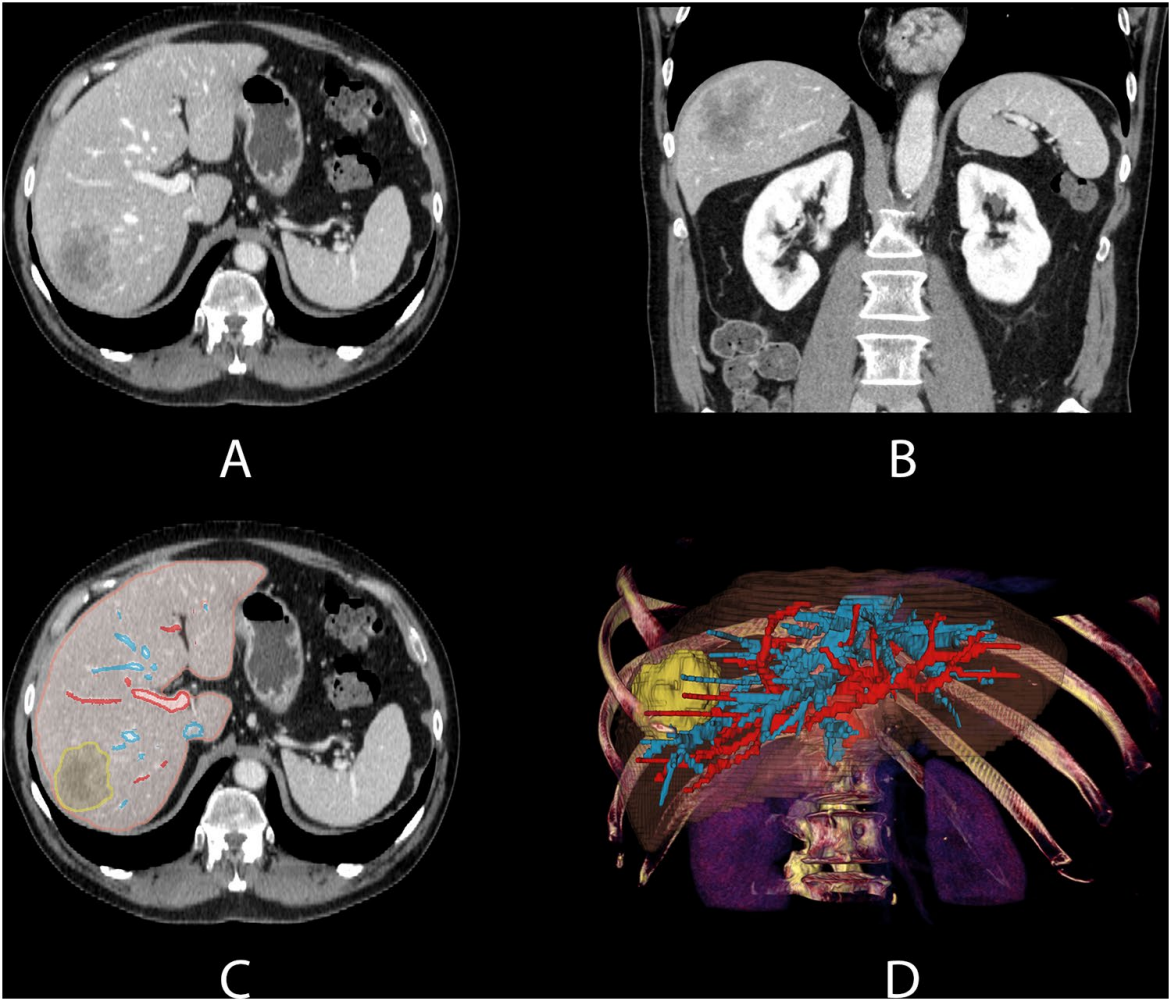
Patient-specific 3D model based on preoperative CT scan

Perioperative management and surgical techniques have been described previously [19]. Standard preoperative investigations included clinical biochemistry, liver ultrasound, contrast-enhanced computed tomography (CT) scans and/or magnetic resonance imaging (MRI) of the thorax and abdomen, and positron emission tomography (PET) scan - if required (cases with suspicion of extrahepatic disease that cannot be confirmed by CT or MRI). In some cases, three-dimensional (3D) patient-specific liver models were created based on pre-operative CT and MRI images and used for virtual resection planning (Fig. 1).

### Preoperative virtual resection planning

Virtual resection planning systems are computer-assisted systems that help surgeons define anatomy, resections and measure properties (e.g., volumetry, distances, safety margins, geometry, etc.) before the actual operation. While LPSLR can be performed using state-of-the-art medical imaging and surgical technology, the use of virtual resection planning systems can provide surgeons with information about the spatial distribution of relevant anatomical structures and the path of planned resection. This information can aid in the decision-making process during the planning and ultimately validate the resection plan.

**Fig. 2:**
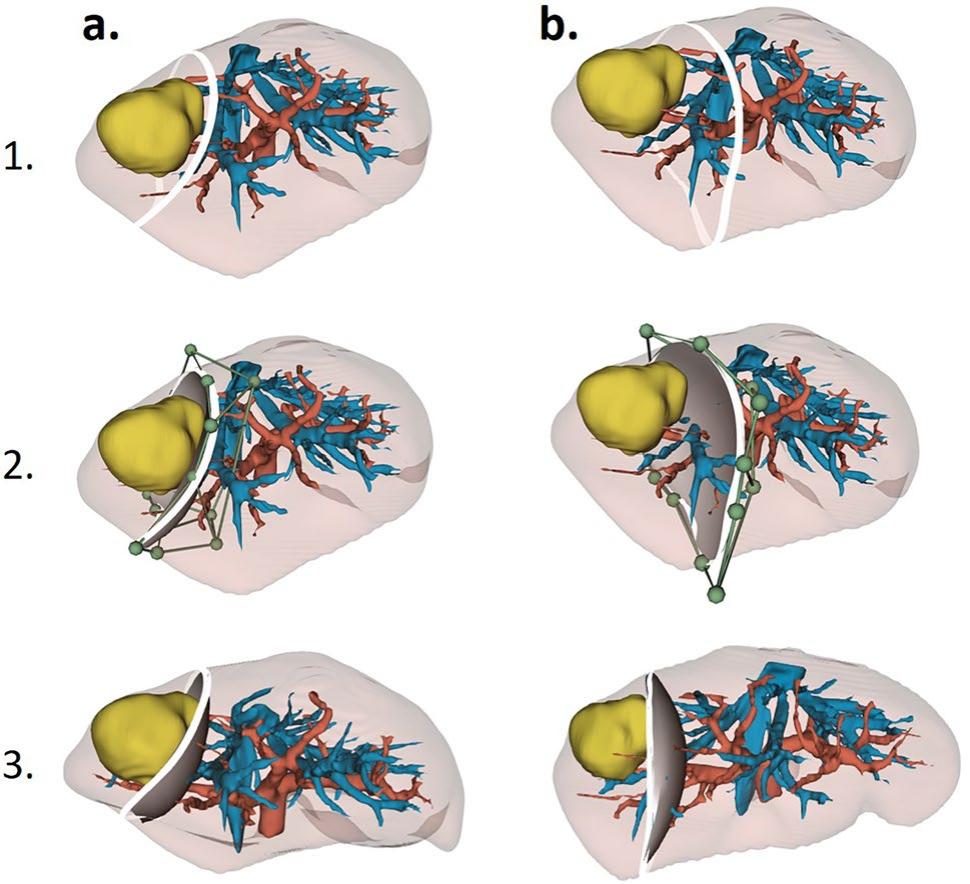
Virtual resection planning steps (1. positioning a resection line and a virtual deformable surface, 2. manipulating the resection plane with control points (green balls) and 3. creating final virtual resection plan). Two proposed resection plans by a computer-assisted system (**a**). an atypical/non anatomic segmentectomy. (**b**). an atypical/non-anatomic bi-segmentectomy, and the final decision is made by the surgeon (Color figure online)

In our workflow, preoperative CT or MRI are first segmented (images are annotated in 3D) and then reconstructed into a 3D patient-specific liver anatomy and pathology model. These 3D models contain liver parenchyma, portal, hepatic veins, and the relevant liver lesions [20–22]. Using a virtual resection planning system, a virtual deformable surface can be placed inside the patient-specific models, enabling the physicians to place and manipulate virtual resections to create a satisfactory resection plan (Fig. 2). Our implementation of a virtual resection planning system uses the software 3D Slicer and a custom-developed software module providing the resection and analysis tools [16, 23]. The necessary preparations (segmentation, 3D model reconstruction, and clinical validation of this information) are performed by a team of computer scientists, biomedical engineers, and clinicians. Surgeons generate and tailor virtual resection plans for individual clinical cases (Fig. 2).

### Definitions and statistics

The 90 days after surgery definition was used to report postoperative mortality, and the Accordion classification was applied to grade postoperative complications [24]. Tumor size was measured following specimen fixation in formaldehyde during the histopathologic analyses of resected specimens. Resection margins were assessed microscopically, and a resection margin of less than 1 mm was defined as positive (R1).

Data are presented as median (range) or mean (SD) and number (percentage). Categorical variables were compared using the Fisher’s exact test or the Chi-square test as appropriate and presented as number (percentage). Non-normally distributed continuous variables were compared using the Mann–Whitney *U* test and are presented as median (range), while normally distributed data are presented as mean (standard deviation [SD]), and Student’s T-test was applied to compare these variables.

Overall survival was estimated from the date of liver resection until death or censoring. Survival probabilities were calculated using the Kaplan–Meier method, and the Log-rank test was applied to compare survival times between the groups. Time-defined survivals are presented in percentage (± standard error). Uni- and multivariable Cox-regression analysis was performed to identify risk factors associated with poor survival. *P*-values less than or equal to 0.05 were considered statistically significant.

SPSS software (IBM Corp. Released 2013. IBM SPSS Statistics for Windows, version 27.0, Armonk, NY, USA: IBM corp.) was used for statistical analysis.

## Results

In total, 587 patients met the inclusion criteria (large tumor group, *n* = 59; and small tumor group, *n* = 528). Patient age and CEA level were higher in those with large tumors. Other baseline characteristics were similar between the groups (Table 1).

**Table 1:** Baseline characteristics

Variable	Large tumors (*n* = 59)	Small tumors (*n* = 528)	*p*-value
Age, year, mean (SD)	69 (9)	66 (11)	**0.026**
Gender, male, *n* (%)	40 (68)	309 (59%)	0.169
BMI, kg/m^2^, mean (SD)	25.7 (4.6)	25.3 (4.1)	0.530
ASA score, *n* (%)			0.132
1/2	31 (52)	331 (63)	
3/4	28 (48)	197 (37)	
Synchronous disease, *n* (%)	37 (63)	313 (59)	0.916
Neoadjuvant chemotherapy, *n* (%)	23 (39)	214 (40)	0.927
CEA level, μg/L, median (range)	8.4 (1–882)	4.6 (1–408)	** < 0.001**
Extrahepatic disease, *n* (%)	10 (17)	104 (20)	0.726
Node positive primary tumor, *n* (%)	38 (64)	297 (56)	0.094

*SD* standard deviation*, BMI* body mass index*, ASA* American Society of Anesthesiologists*, CEA* carcinoembryonic antigen

Median tumor size was 60 mm (range, 50–110) in the large tumor group and 21 mm (range, 3 to 48) in the small tumor group (*p* < 0.001) (Table 2).

**Table 2:** Perioperative outcomes

Variable	Large tumors (*n* = 59)	Small tumors (*n* = 528)	*p*-value
Tumor size, mm, median (range)	60 (50 to 110)	21 (3 to 48)	** < 0.001**
Localization of resection, *n* (%)			0.256
AL segments	26 (44)	269 (51)	
PS segments	25 (42)	168 (32)	
Mixed	8 (14)	91 (17)	
Operation time, min, mean (SD)	160 (69)	142 (75)	0.091
Blood loss, ml, median (range)	500 (30 to 3500)	200 (20 to 4400)	** < 0.001**
Combination with ablation, *n* (%)	2 (3)	41 (8)	0.297
Simultaneous procedures, *n* (%)	14 (24)	78 (15)	0.073
Cholecystectomy	5	37	
Colorectal surgery	0	14	
Abdominal lymph node dissection	3	5	
Adrenalectomy	1	4	
Other	5	18	
Conversion to laparotomy, *n* (%)	2 (3.4%)	14 (2.7%)	0.761
Morbidity (≥ Grade 2), *n* (%)	12 (20%)	88 (17%)	0.498
90 days mortality, *n* (%)	0	2 (0.4%)	1.000
Postoperative stay, days (range)	3 (1 to 25)	2 (1 to 35)	0.139
Number of lesions, median (range)	1 (1 to 4)	1 (1 to 7)	0.766
R1 (< 1 mm) resections, *n* (%)	14 (24)	117 (22)	0.586
Involved resection margin, *n* (%)	8 (14)	55 (10)	0.303

*AL* antero-lateral*, PS* postero-superior

In the large tumor group, 56% of the patients had resection in the postero-superior segments (technically major resections), versus 49% of patients in the small tumor group. Fourteen (24%) patients in the large tumor group and 78 (15%) in the small tumor group had other simultaneous abdominal procedures (*p* = 0.073). Operation time and conversion rate were similar, while median blood loss was higher in the large tumor group (500 ml vs. 200 ml, *p* < 0.001). Other perioperative outcomes, including postoperative morbidity and mortality, were similar. No difference in positive resection margins was found between the groups (Table 2).

**Fig. 3:**
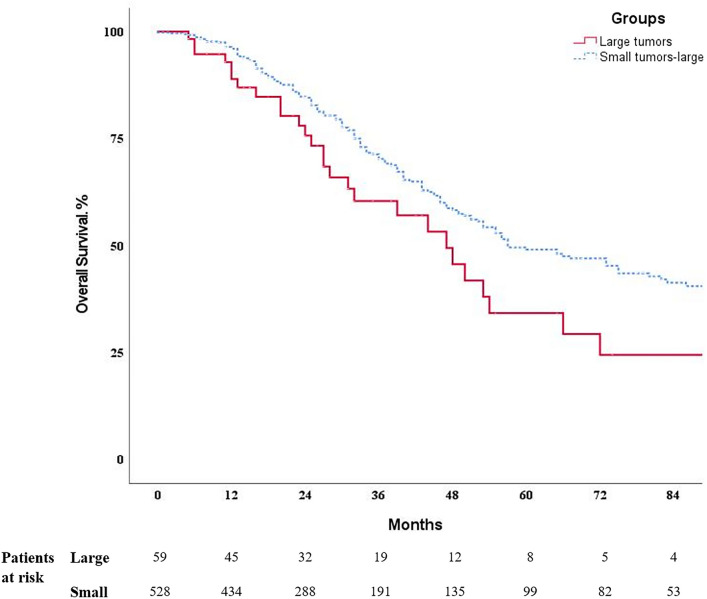
Kaplan–Meier survival curves for patients with large and small CRLM

Patients in the large tumor group had significantly shorter median overall survival, 47 (95%CI 35 to 59) months versus 57 (95%CI 46 to 68) months) (*p* = 0.027). 5 year overall survival was 34% (± 8.6) in the large tumor group and 49% (± 3.1) in the small tumor group (Table 3; Fig. 3). However, in the multivariable Cox-regression analysis, tumor size did not impact survival, unlike parameters such as patients’ age, ASA score, CEA level, presence of extrahepatic disease at liver surgery, and positive lymph nodes in the primary tumor that were independent predictors for poor overall survival (Table 4).

**Table 3:** Overall survival rates

	Large tumors (*n* = 59)	Small tumors (*n* = 528)	*p*-value
Median OS, months	47 (95% CI 35–59)	57 (95% CI 46–68)	**0.027**
1-year	89% (± 4.3)	96% (± 1.0)	
3-year	60% (± 7.5)	70% (± 2.4)	
5-year	34% (± 8.6)	49% (± 3.1)	

*OS* overall survival*, CI* confidence interval

**Table 4:** Risk factors for poor overall survival (Cox-regression analysis)

Variable	Univariable		Multivariable	
	HR	*p*-value	HR	*p*-value
Age, per year	1.02 (1.01 to 1.04)	** < 0.001**	1.03 (1.01 to 1.05)	**0.001**
Gender (male)	1.12 (0.85 to 1.48)	0.404		
BMI, kg/m^2^	1.00 (0.97 to 1.04)	0.768		
ASA score (3/4)	1.68 (1.28 to 2.20)	** < 0.001**	2.44 (1.65 to 3.59)	** < 0.001**
Synchronous disease	1.06 (0.81 to 1.39)	0.671		
CEA level, ng/mL	1.002 (1.000 to 1.004)	**0.014**	1.003 (1.001 to 1.005)	**0.004**
No neoadjuvant chemo	0.878 (0.67 to 1.15)	0.355		
Extrahepatic disease	1.92 (1.35 to 2.74)	** < 0.001**	1.66 (1.01 to 2.74)	**0.047**
N + primary	1.88 (1.35 to 2.62)	** < 0.001**	1.90 (1.21 to 2.98)	**0.005**
Number of tumors	1.18 (1.04 to 1.34)	**0.009**	1.10 (0.92 to 1.31)	0.318
Size of tumor (cm)	1.15 (1.06 to 1.24)	** < 0.001**	1.10 (0.99 to 1.22)	0.07
R1 resection	1.81 (1.31 to 2.51)	** < 0.001**	1.80 (0.98 to 3.31)	0.058
Involved margin	1.63 (1.11 to 2.41)	**0.013**	1.01 (0.47 to 2.15)	0.976

*HR* hazard ratio

The preoperative virtual resection planning method was used in some of the more advanced cases included in this study during the last years and thus not systematically implemented in routine practice throughout the study period. Therefore, it is not presented as a variable to evaluate its impact on the surgical outcomes but as a tool for verification of the resection strategy and decision support.

## Discussion

Laparoscopic approach to resect large liver lesions remains debatable and may still be a relative contraindication in many centers. Parenchyma-sparing liver resection performed by laparoscopic access in patients with large tumors can be technically challenging and is preserved for surgeons with extensive experience. The findings of the current analysis show that LPSLR for patients with large CRLM can achieve satisfactory results, similar to those with small lesions. It was associated with higher blood loss, while other perioperative outcomes were similar.

The parenchyma-sparing strategy for CRLM has shown its advantages and has been widely used [25, 26]. These resections are associated with decreased morbidity and increased salvageability and may improve the patients’ survival by facilitating future liver resections in case of liver recurrences [27–29]. In the report from Torzilli and colleagues, the authors distinct the parenchyma-sparing liver surgery as a minimally invasive surgery in a hepatic-centered perspective even if the surgery is performed by open approach [30].

In expert centers as well as in our center, laparoscopic liver surgery has been safely adopted and is used to perform numerous types of liver resections^1^. In a systematic review and meta-analysis by Kalil et al., the laparoscopic approach to perform parenchyma-sparing liver resections was defined as “maximally minimally invasive” surgery of the liver [31]. However, large liver malignancies to be removed by laparoscopic approach remains questionable, and the current data is limited by the case series with a relatively small number of patients [32, 33]. To the best of our knowledge, our report consists of the largest series focusing on laparoscopic liver resection for large CRLM and is the first study reporting parenchyma-sparing strategy for these patients. Comparison with the group of patients with smaller metastases shows that laparoscopic resection of large liver tumors in expert hands can achieve similar surgical outcomes. The higher blood loss seen in this series is in line with the previous studies on laparoscopic liver resection for large liver tumors [32, 34]. The worse overall survival in the large tumor group was somehow expected since the size of the tumor is a prognostic factor and has been included in clinical scoring systems [35, 36]. However, in this series, the tumor size did not significantly impact patients' survival in multivariable analysis (Table 4). It might be explained by the higher median value of the CEA level in patients with large tumors, which might have adjusted the impact of the tumor size.

LPSLR has become a standard surgical method in our center and is our preferred approach, especially in patients with CRLM, and it is preferred whenever possible. However, computer-assisted systems for patient-specific anatomy visualization and surgery planning could further improve the procedure. Through medical image segmentation and reconstruction techniques, 3D patient-specific liver models can help in surgery planning, especially in challenging cases, such as large tumors, tumors located in the “difficult” segments, deep located tumors, and tumors with close proximity to the major vessels. Moreover, resection planning using these imaging advancements and taking into account both inflow and outflow to the resection area can provide surgeons a better understanding of individual patient liver anatomy, tumor location, and its relation to the vessels, as well as a precise trajectory of the resection plane.

The current analysis has several limitations. Firstly, information bias is possibly present due to the retrospective nature of this analysis. The presented computer-assisted resection planning method was not presented as a variable, and we could not evaluate its impact. Further investigations are in process, and more results will be available in the future. False-negative findings are possible when comparing the groups, caused by the significant difference in the number of patients in the groups, which is another weakness of this study.

## Conclusion

Based on our experience and the current analysis results, we may conclude that laparoscopic liver surgery is safe and provides good surgical and oncologic outcomes even for challenging tumors. Laparoscopic parenchyma-sparing liver resections should be preferred whenever technically possible. The continuous advancements in medical technologies can potentially improve these procedures.
